# Editorial: Leadership, diversity and inclusion in organization

**DOI:** 10.3389/fpsyg.2023.1184939

**Published:** 2023-06-01

**Authors:** Daniel Roque Gomes, Neuza Ribeiro, Kamila Ludwikowska

**Affiliations:** ^1^Escola Superior de Educação de Coimbra, Instituto Politécnico de Coimbra, Coimbra, Portugal; ^2^Centre of Applied Research in Management and Economics, Polytechnic Institute of Leiria, Leiria, Portugal; ^3^Faculty of Management, Wrocław University of Science and Technology, Wrocław, Poland

**Keywords:** editorial, leadership, authentic leadership, diversity and inclusion, virtuous lea

The issues surrounding the human side of managing organizations are quite varied if considering the plethora of determinants, contexts or processes that have been under consideration by researchers (e.g., Duarte et al., 2019; Koutsimani et al., 2019; Ribeiro et al., 2020; Herrera and Heras-Rosas, 2021). Leadership in organizations was, is and will continue to be a key issue for determining the functioning of organizations, regardless of their genesis or purposes of action (e.g., Semedo et al., 2018; Li et al., 2022). The starting point of this Research Topic has resided in the recognition that today's society is undergoing through deep and significant transformations, occurring at an unprecedented speed and producing severe impacts over the functioning of organizations. Within this framework, it is possible to find social transformations related to the strengthening of social and work equality. A good example over this matter is the issues of gender equality or the acceptance of diversity as a desirable societal value, along with the value of inclusion or the promotion of individual and collective freedoms. It is also within this context that we find grounds for considering the application of social values oriented toward inclusion and equal treatment of minorities as a socially relevant issue with impactful consequences in the context of work organization and the functioning of the world of organizations.

Considering this context, we believe that it was pertinent to organize a Research Topic aimed at positioning these issues within the scope of the effects of organizational leadership in promoting more inclusive and egalitarian work environments, along with the identification of the main determinants, processes and contexts supporting these issues. Guided by the reasons already portrayed, and which motivated us to organize this Research Topic, organizing the call for papers for the topic has gained proportion when we realized that the existing literature, while recognizing the “business case for diversity,” does not produce convincing answers to the questions raised. This Research Topic produced a set of 14 articles that deal with different and complementary aspects of the nature of the discussion proposed to researchers.

The study by Li et al. “*Perceived overqualification at work: implications for voice towards peers and creative performance*” discusses the importance of voice toward peers in the context of stimulating creativity in organizations and the relevance of considering the team background in producing relevant organizational results. In another framework, the study by Bajcar and Babiak offers the empirical validation of the Multifactor Leadership Questionnaire (MLQ 5X Short) in the European context, namely in Poland, providing an important contribution to the future application of the instrument when studying organizational phenomena suitable for your application. Complementarily, the study by Yin and Liu “*The relationship between empowering leadership and radical creativity*” discusses the centrality of empowering leadership in the production of effects on the creativity of workers, verifying the positive indirect influence of empowering leadership via the mediation effects of job control and willingness to take risks.

Following a similar theoretical alignment, the study by Jing et al. “*The influence of empowering team leadership on employees' innovation passion in high-tech enterprises*” discussed the impact of empowering team leadership in stimulating innovation passion among workers, finding a cross-level direct positive and indirect positive influence on innovation indicators. The study by Zhu and Chen “*Work-to-family effects of inclusive leadership: the roles of work-to-family positive spillover and complementary values*,” investigated the impact of Inclusive Leadership on employees' wor-to-family positive spillover along with the positive impact on the family performance enhancement.

Another important study was presented by Wang et al. “*Can proactive confessing obtain your embrace? Exploring for leader's pro-social rule-breaking consequences based on a self-verification perspective*” in which they highlighted the relationship between leader pro-social rule breaking and leader feedback-seeking, which in turn is related to employee upward voice. Another equally relevant study was developed by Özgenel et al. “*The mediator role of organizational justice in the relationship between school principals' agile leadership characteristics and teachers' job satisfaction*,” in which the authors identified the important relationship between Schools Principals' agile leadership characteristics are associated with teachers' job satisfaction.

Liu and Liu, in the study “*The implications of inefficient markets for executive pay comparison: the case of China and Poland*,” the authors emphasize the importance of the impact of pay comparison among executive leaders. Lee et al. have presented an opinion article entitled “*Is authentic leadership always good for employers? The perspective of time management*,” and presenting a reflection on the impact of this type of leadership on production indicators in organizations, supporting the consolidation of knowledge on the subject.

Another article equally considered in this Research Topic was developed by Choi et al. “*What hinders team innovation performance? Three-way interaction of destructive leadership, intra-team conflict, and organizational diversity*,” in which the authors clarify the impact of destructive leadership on team innovation performance, and identify the conditions that contribute to the exponentiation of the relationship. An important contribution to be noted in the Research Topic was the one made by Kusku et al. entitled “*Beyond the three monkeys of workforce diversity: who hears sees and speaks up?*” and in which the authors explain the differences between those who remain indifferent to diversity works and those how see, speak and hear about them.

In another perspective, the study by Teofilus et al. “*Managing organizational inertia: indonesian family business perspective*” provides interesting insights into the role of different actors within the framework of the determinants of inertia in organizations and associated aspects of organizational performance, and about the role of Empowering Leadership in this relationship. Another relevant research to consider under the topic considered here refers to the study carried out by Sajadi and Vandenberghe “*Supervisors' social dominance orientation, nation-based Exchange relationships, and team-level outcomes*,” in which it is explored how the orientation of social dominance of supervisors relates to relational conflicts and commitment. This Research Topic was also enriched with a brief research report article by Zhao and Zhou, entitled “*Analysis of the turnover tendencies of college teachers from the perspective of psychology*,” in which the problem of college teachers' turnover intention is discussed, and focusing on factors that contribute to understanding the underlying psychological mechanisms of teachers' resignation.

Once all the contributions received for this Research Topic have been considered, we conclude that a wide range of cumulative perspectives have been produced over the theme that motivated the organization of the topic. Indeed, it is visible that the topics brought up for discussion under the aegis of the theme of Leadership, Diversity and Inclusion in Organizations was rich and fruitful. However, the area of studies on Leadership, Diversity and Inclusion is still recent in the literature, so we hope that readers and those interested in the themes portrayed here will feel motivated and compelled to produce new research on the subject, supporting the scientific community to better respond in the face of current and future challenges related to the exercise of Leadership in Organizations.

## Author contributions

All authors listed have made a substantial, direct, and intellectual contribution to the work and approved it for publication.
